# Lysozyme association with circulating RNA, extracellular vesicles, and chronic stress

**DOI:** 10.1016/j.bbacli.2016.12.003

**Published:** 2016-12-20

**Authors:** Sarah K. Abey, Yuana Yuana, Paule V. Joseph, Natnael D. Kenea, Nicolaas H. Fourie, LeeAnne B. Sherwin, Gregory E. Gonye, Paul A. Smyser, Erin S. Stempinski, Christina M. Boulineaux, Kristen R. Weaver, Christopher K.E. Bleck, Wendy A. Henderson

**Affiliations:** a Digestive Disorders Unit, Division of Intramural Research, National Institutes of Health, Department of Health and Human Services, Bethesda, MD, USA; b Image Sciences Institute, Division of Imaging, University Medical Centre Utrecht, Netherlands; c NanoString Technologies, Seattle, WA, USA; d The Pennsylvania State University, College of Medicine, Hershey, PA, USA; e Electron Microscopy Core Facility, National Heart, Lung, and Blood Institute, National Institutes of Health, Department of Health and Human Services, Bethesda, MD, USA

**Keywords:** EVs, Extracellular vesicles, CD9, cluster of differentiation 9, CXCL12, Chemokine (C-X-C motif) ligand 12, IL, interleukin, PD-L1, programmed death ligand 1, NT5E, 5′-Nucleotidase Ecto, CD9, lysozyme, wound, inflammation, stress, CXCL12

## Abstract

**Background:**

Stress has demonstrated effects on inflammation though underlying cell-cell communication mechanisms remain unclear. We hypothesize that circulating RNAs and extracellular vesicles (EVs) in patients with chronic stress contain signals with functional roles in cell repair.

**Methods:**

Blood transcriptome from patients with Irritable Bowel Syndrome versus controls were compared to identify signaling pathways and effectors. Plasma EVs were isolated (size-exclusion chromatography) and characterized for effectors' presence (immunogold labelling-electron microscopy). Based on transcriptome pathways and EV-labelling, lysozyme's effects on cell migration were tested in human colon epithelial CRL-1790 cells and compared to the effects of CXCL12, a migration inducer (wound assay). The effect of lysozyme on immune-linked mRNA and protein levels in cells which survived following serum starvation and scratch wound were investigated (NanoString).

**Results:**

Blood transcriptomes revealed pyridoxal 5’phosphate salvage, pyrimidine ribonucleotides salvage pathways, atherosclerosis, and cell movement signaling with membrane CD9 and extracellular lysozyme as effectors. Plasma EVs showed labelling with CD9, mucins, and lysozyme. This is the first identification of lysozyme on plasma EVs. In CRL-1790 cells, lysozyme induced migration and repaired scratch wound as well as CXCL12. Immune mRNA and protein expressions were altered in cells which survived following serum starvation and scratch wound, with or without lysozyme in serum-free media post-wounding: CD9, IL8, IL6 mRNAs and CD9, NT5E, PD-L1 proteins.

**Conclusions:**

Repair and inflammatory signals are identified in plasma EVs and circulating RNAs in chronic stress. Registered clinicaltrials.gov #NCT00824941

**General significance:**

This study highlights the role of circulating RNAs and EVs in stress.

## Highlights

• Inflammatory pathways are predicted by blood transcriptome.
• Plasma EVs from patients and controls show labelling with lysozyme, CD9, and mucins.
• Lysozyme induces cell migration in response to wound *in vitro.*
• Lysozyme alters immune-linked mRNA and protein cellular levels in response to wound.
• Circulating RNAs and EVs contain signals with roles in cell migration and repair.

## 1 Introduction

Responses to wound or injury consist of multicellular signaling networks of immune-linked pathways involving cell migration and regeneration [1], [2]. These responses are thought to be delayed under conditions of stress, such as prolonged metabolic stress or psychosocial stress [1], [3]. Although signaling pathways driven by cytokines such as the transforming growth factor beta (TGFβ) and interleukin 1 (IL1) are now known to play a role in cellular responses to injury, the molecular signals for cell-cell communication in the multicellular signaling network are still poorly understood [4], [5]. Besides cytokines, extracellular vesicles (EVs) were recently found to be released during the wounding of human epithelial cells *in vitro* [6]. Since their first description in 1946 as “platelet-derived particles in normal plasma”, EVs found circulating in various types of human body fluids, such as plasma and urine, are gaining significance as a form of vehicle for cell-cell communication signals [7], [8], [9], [10]. EV-associated signals in the circulation are diverse in terms of biochemical properties, encompassing proteins, lipids, and genetic materials, and like cytokines, they have functional implications in several major pathological processes such as metastasis [11] and fibrosis [12]. Like EVs, circulating RNAs are also proposed to have functional significance beyond utilization as biomarkers in diseases [13]. Circulating RNAs were shown to change in their levels post-injury in correspondence to alterations in specific biological pathways post-injury [14]. Thus, given the significance of circulating biological signals, identifying and elucidating the potential functions of such signals are necessary for our understanding of complex multicellular networks, such as those employed by cells in response to injury during periods of stress.

In the gastrointestinal (GI) tract, intestinal epithelial cells undergo continuous processes of repair and regeneration at high turnover rates to maintain the gut barrier [15]. Intestinal epithelial cells secrete vesicles [16] and EVs tagged by the membrane protein CD9 are suggested to be implicated in repair and regeneration [6]. Innate immune guardians and antimicrobial proteins, such as lysozyme, and mucus layer proteins, such as mucins, reside in the intestinal mucosa and contribute to the maintenance of intestinal homeostasis [17], [18]. Because of their roles in immune pathways, both are thought to contribute to the regulated processes of cellular reprogramming after injury as well as translocation of microbes or microbial antigens across the gut barrier [18], [19]. Recently, lysozyme and mucins were found in urine EV fractions from healthy individuals [20]. However, although EVs in the circulations are known to carry mucins as indicators of intestinal epithelial origins [21], the biological association between lysozyme with circulating EVs has only been studied as part of its utilization as a model to understand amyloid fibril formations [22]. Lysozyme is a basic protein capable of interacting with negatively charged phospholipid bilayers and inducing the aggregation of phospholipid vesicles at low or neutral pH [23], [24], [25]. More recently, endogenous lysozyme was found to modulate the composition of EV-associated RNA during inflammation, suggesting its role in cell-cell communication signaling during inflammatory response [26]. Supporting this role in inflammation related to the GI system, lysozyme co-localizes with lipopolysaccharide binding protein (LBP), the binding partner of lipopolysaccharides (LPS), in mouse intestine [27]. LBP is a liver-derived molecule which is induced by the translocation of bacterial LPS into the circulation and can initiate an inflammatory signaling cascade through toll like receptors or TLRs [28], [29]. Recently, lysozyme levels were found to be upregulated in histological staining of various inflammatory GI diseases [30]. Thus, lysozyme is a plausible candidate for cell-cell communication signaling during cellular response to injury, and part of this role may be afforded by its affinity for negatively charged EVs.

We hypothesized that circulating RNAs and plasma EVs from patients with chronic stress contained signals with functional implications on cell repair or injury response pathways. Patients who had Irritable Bowel Syndrome (IBS) according to established Rome III criteria were characterized as IBS-D, for chronic diarrhea, or IBS-C, for chronic constipation [31]. Chronic stress is a co-morbidity of IBS as we and others have previously reported [32], [33], and the role of various stressors or injury in the development of IBS, including physical injury or interoceptive stressors such as surgery and enteric infection, is thought to be associated with symptom chronicity in this functional GI disorder [34], [35]. An inflammatory component of IBS pathophysiology has been suggested in relation to injury in the GI tract [36]. In our IBS cohort, we have observed an altered intestinal permeability specific to the colon [37], which may reflect molecular changes in the colon-associated cells within the epithelial barrier of these patients. More recently, we reported an altered microbiome composition reflecting a reduction in the number of bacterial families commonly known to produce metabolites required for intestinal health, such as the short chain fatty acids, in IBS patients versus controls [38]. This further illustrated a possible epithelial homeostatic effects mediated by microbial alterations accompanying the suspected chronic GI barrier damage in IBS [38]. Thus, understanding the signaling network associated with IBS could shed light into the chronic stress pathophysiology and the observed GI symptoms in these patients.

In order to identify chronic stress-associated signaling network, we first analyzed the circulating RNA expression (transcriptome) profiles of IBS patients versus controls. We constructed a signaling network based on this transcriptome analysis. Next, plasma EVs from representative patients and controls were isolated and labelled for surface signals, according to information from the transcriptome-based signaling network. Immunolabelling of lysozyme, in addition to CD9 and mucins, was found on intact plasma EVs. Because another antimicrobial peptide, the human beta-defensin, was recently found to induce intestinal epithelial cell migration [39], and because cell migration was revealed among the top pathways constructed by our IBS cohort's transcriptome analysis, we concentrated subsequent efforts on investigating the functional relevance of lysozyme in this context. The primary, non-transformed diploidic human colon CRL-1790 cell line was chosen based on its epithelial characteristics [40], lack of tumor-associated keratin [41], and reports of the low endogenous level of beta-catenin expression related to its utilization as a non-migratory control for studies of colorectal adenocarcinoma metastasis pathophysiology [42]. We investigated cell migration in response to scratch wound in CRL-1790 cells incubated with or without lysozyme. Finally, we examined endogenous alterations in the RNA and protein levels of CRL-1790 cells which survived at the time when collective cell migration was observed (8 h). Global cellular RNAs and proteins related to immune and inflammatory functions in post-wound surviving cells were examined using the nCounter® PanCancer Immune Panel (Nanostring). During the processes of repair following wound or injury only surviving cells were analyzed by this Panel. The RNA and protein analyses reflect immune-linked molecular reprogramming which occurred in CRL-1790 cells, when lysozyme was present compared to when it was absent extracellularly.

## 2 Materials and methods

### 2.1 Study subjects

Participants (age 13–45 years) who were normal weight or overweight, with or without history of chronic (≥ 6 months) GI symptoms according to Rome III criteria for IBS were recruited under a natural history protocol at Hatfield Clinical Research Center, National Institutes of Health (NIH), between January 2009 and October 2013. The study was approved by the NIH Institutional Review Board and complied with the Declaration of Helsinki with voluntary written informed consent from all participants included in the study (clinicaltrials.gov #NCT00824941). Individuals with organic GI disease history (e.g., inflammatory bowel disease, celiac disease, biliary disorders, bowel resection) or taking daily medications for GI symptoms (e.g., 5-HT3 antagonists, laxatives, anti-diarrheal, antispasmodics) or serotonin-altering medications (e.g., SSRI, catecholamines, cortisol-excluding inhaled corticosteroids) were excluded. Likewise, all patients are afebrile and assessed in real time for recent or active infections including complete blood count and negative urine and stool cultures. Biological samples (blood, plasma) were collected during two-day outpatient visits within the same week. Male and female participants with IBS (*n* = 28) were matched with healthy controls (*n* = 62).

### 2.2 Blood RNA collection and gene expression profiling

Peripheral whole blood was collected in PAXgene® RNA tubes and total RNA was isolated with PAXgene® Blood miRNA kit as per the manufacturer's instructions. RNA quantity, purity, and integrity were assessed by a NanoDropTM 1000 spectrophotometer (Wilmington, DE) or by 2100 Bioanalyzer using the RNA 6000 Nano LabChip kit (Agilent Technologies, Santa Clara, CA). All samples yielded high quality score (RIN > 9). Whole genome gene-expression was measured using GeneChip Human Genome U133 + 2.0 arrays (Affymetrix). Double-stranded cDNA, was synthesized from 10 ng total RNA followed by a linear isothermal amplification to produce single-strand cDNA, which was direct labeled by biotin using Ovation Whole Blood Solution kit (NuGEN technologies, Inc., San Carlos, CA) following manufacturer's protocol. Biotinylated cDNA (4.4 μg) from each sample was mixed with the control buffer and hybridized on each Human Genome U133 plus 2.0 array (Affymetrix, Santa Clara, CA) at 45 °C for 16 h in a GeneChip hybridization oven at 60 rpm. GeneChip arrays were washed on Fluidics Station 450 using manufacturer's recommended scripts and scanned on GeneChip Scanner 3000 (Affymetrix, Santa Clara, CA). GeneChip Command Console (AGCC 3.0, Affymetrix) was used to scan the images and for data acquisition. For comparison between arrays, a global scaling factor (target signal set to 500) was used across arrays to minimize the variables caused by sample preparation, hybridization, staining or from different manufactured lots of arrays. Microarray quality control was evaluated for each array via examination of background, noise, average signal, % present, ratio of signal values for probe sets that represented the 5′ and 3′ ends of actin and glyceraldehyde-3-phosphate dehydrogenase transcripts, and total signal for probesets for BioB, BioC, BioD and CreX. Gene expression data were analyzed using multi-way mixed ANOVA model by Partek Genomic Suite software (Partek, Inc., St. Louis, MO). Statistical significance was set at *p* ≤ 0.05 and 1.5-fold change boundary in expression was required to accept alteration in expression as significant. Microarrays were processed at the Laboratory of Molecular Technology, National Cancer Institute (NIH, Frederick, MD), following standard operating protocol to minimize non-biologic technical bias.

### 2.3 In silico analysis of transcriptome-based pathways

Ingenuity Pathway Analysis (IPA) and **D**atabase for **A**nnotation, **V**isualization and **I**ntegrated **D**iscovery (**DAVID**) v6.8 [43] were utilized to create functional biological network of signaling pathways associated with gene expression profile of IBS patients versus controls. Only genes with fold difference above the 1.5-fold change boundary and *p* ≤ 0.05 were used as input. A model of cellular signaling networks from differential gene expression profiling of patients versus controls was created by IPA. Top canonical pathways, top molecular and cellular functions, and top networks IPA were retrieved as results. In addition, functional annotation of genes from this differential profiling was performed using DAVID. DAVID's functional annotation tool identifies clusters of genes from annotation categories that are differentially expressed between the groups. Gene ontology (GO) terms under the categories of biological processes, cellular components, and molecular functions with top three counts of genes included during DAVID analysis were retrieved.

### 2.4 LBP

Serum levels of LBP were measured by enzyme-linked immunosorbent assay (ELISA) per manufacturer's instructions (Cell Sciences, Canton, MA).

### 2.5 Statistical analysis

Clinical data statistical analyses were completed using Statistica, Version 12 (StatSoft, Tulsa, OK), including parametric *t*-tests and non-parametric Mann-Whitney tests and statistical significance was defined for *p* value ≤ 0.05. For parametric tests, means ± standard deviations were reported, whereas for non-parametric tests, medians ± interquartile ranges were reported. *In vitro* experiments were performed with at least three replicates of samples and means ± standard deviations were reported. Statistical analyses for nCounter mRNA and protein data were performed as described under “nCounter® PanCancer Immune Panel (NanoString)” methods.

### 2.6 Plasma EV isolation

Peripheral blood was collected in citrate tubes (BD Biosciences), centrifuged at 2000 X g at 4 °C for 15 min, and supernatants were stored as aliquots at − 80 °C until isolation. Aliquots were thawed at 37 °C and briefly vortexed. For EV isolation, 750 μL plasma was subjected to size-exclusion chromatography by qEV Size Exclusion Columns (Izon Science Ltd., Oxford, UK). Briefly, the qEV columns were fastened using clamps onto the support stand. These columns were levelled before use and rinsed at least with 10 mL of PBS containing sodium chloride (154 mmol/L), di-sodium hydrogen phosphate dihydrate (1.24 mmol/L) and sodium dihydrogen phosphate dihydrate (0.21 mmol/L) adjusted at pH 7.45 and filtered through a 0.22 μm syringe disc filter (PVDF membrane, Merck Millipore). Sample was pipetted onto the column, followed by elution with PBS supplemented with 0.32% sodium citrate (Merck Millipore). The first 4 mL of eluate was discarded. Subsequent 1 mL of eluate containing EVs (fractions 9–10) was collected. The isolated EV sample was snap frozen in liquid nitrogen and stored at − 80 °C.

### 2.7 EV immuno-labelling and imaging by transmission electron microscopy

Glow-discharged 300 mesh formvar and carbon coated nickel grids (Electron Microscopy Sciences, Hatfield, PA) were floated on 10 μL drops of sample and incubated for 7 min at room temperature (RT). The grids were washed three times using PBS (pH 7.45, 0.22 μm filtered). Next, the grids were incubated in blocking solution (Aurion, Wageningen, The Netherlands) for 30 min at RT followed by washing three times with 0.1% BSA-c (Aurion). After the last washing step, excess liquid on the grids was removed by blotting with filter paper. For immuno-labelling, the grids were incubated for 1 h (hr) with 10 μL of mouse anti-human primary antibody. These were mouse monoclonal anti-human CD9 clone M-L13 (dilution 1:50, BD Biosciences, San Jose, CA), mouse monoclonal anti-human lysozyme clone BGN/06/961 (dilution 1:5, Abcam, Cambridge, MA), mouse monoclonal anti-human MUC1 clone HMFG1 (dilution 1:5, Abcam), or mouse monoclonal anti-human MUC2 clone 996/1 (dilution 1:5, Abcam). Negative controls mouse IgG1, ĸ isotype control clone × 40 (BD Biosciences) or mouse IgG2a, ĸ isotype control clone G155–178 (BD Biosciences) at the same concentration as the primary antibody were used. Washing steps were done at least four times with 0.1% BSA-c to remove the unbound antibody. Afterwards, the grids were incubated with 30 μL goat anti mouse IgG secondary antibody labelled with 10-nm gold particles (BBI Solutions, Cardiff, UK) for 1 h. The grids were again washed three times with 0.1% BSA-c followed by three times washing with PBS. To fix the labelled sample, the grid was incubated for 5 min with 2% glutaraldehyde and washed twice with PBS. Next, the grids were transferred to 1.75% uranyl acetate (*w*/*v*) drops for negative staining. Dry-blotted grids were imaged by JEM1200 EX-II transmission electron microscope (JEOL USA) equipped with an AMT XR-60 digital camera (Advanced Microscopy Techniques).

### 2.8 Cell culture

The human fetal colon epithelial cell line CRL-1790 was obtained from American Type Culture Collection (ATCC, Manassas, VA) and maintained in manufacturer's recommended Eagle's Essential Minimal Media (EMEM) supplemented with 10% fetal bovine serum (ATCC). Cells were maintained under a humidified 5% CO_2_ atmosphere at 37 °C.

### 2.9 Wound healing scratch assay and fluorometric migration assay

Cells were grown as monolayers in triplicates in 6-well plates (7 × 10^5^/well) until confluent. Cells were then pre-treated with serum-free media containing 200 μg/mL Bovine Serum Albumin (BSA; New England Biolabs, Ipswich, MA) overnight. The next day, cell monolayer was scratched using a sterile micropipette tip. After washes with serum-free media, fresh serum-free media containing only BSA (“serum-free”), BSA with purified chicken white egg lysozyme (10 μg/mL; Sigma-Aldrich, St. Louis, MO) or CXCL12 or Stromal Derived Factor-1 (10 ng/mL; SDF-1; ProspecBio, East Brunswick, NJ), or 10% FBS was added. For preliminary experiments, cells were incubated for 4, 8, 12, or 24 h. For Fig. 3, cells were incubated for 8 h at which time migration into scratch wound gaps was observed in lysozyme-treated and CXCL12-treated cells. The images of the wounded area were captured immediately after the scratch (0 h) or at 8 h. Image capture and processing (100 μm scale shown) were done using Leica microscope, Olympus camera (Olympus Scientific, Waltham, MA), and CellSens software.

Fluorometric assay was performed according to manufacturer's instructions (ECM509 QCM™ 24-Well Fluorimetric Cell Migration Assay, EMD Millipore, Billerica, MA). Briefly, CRL-1790 was plated to 80% confluency and incubated in serum-free media overnight. The next day, cells were harvested and resuspended in serum-free media containing 200 μg/mL BSA. Cells were seeded at 3 × 10^5^ cells/300 μL media in 24-well, 8 μm pore size, Boyden chamber inserts. Media containing lysozyme (10 μg/mL) or CXCL12 (10 ng/mL) were added into the outer chamber. Cells were incubated for 24 h at 37 °C/5% CO_2_, followed by detachment, lysis, and binding to CyQUANT GR Dye. Automated fluorescence readings were performed in 96-well plates with 480/520 nm filters using SpectraMax M3 Microplate Reader (Molecular Devices, Sunnyvale, CA).

### 2.10 nCounter PanCancer Immune Panel (NanoString)

For NanoString sample preparation, CRL-1790 cells were collected at 0 h or 8 h (incubated in serum-free media with or without lysozyme) after wounding, as described under “wound healing scratch assay.” Cells collected at these time points were cryo-protected in EMEM/10% FBS containing 5% DMSO. On the day of assay, cells were thawed and incubated for 2–3 h in EMEM/10% FBS at 37 °C 5% CO_2_. Following this period, cells were collected, counted using Cellometer (Nexcelom Bioscience, Lawrence, MA), and percent live cells (viability) was determined. Based on the cell counts, 240,000 live cells were pelleted and re-dissolved in 400 μL 1 × PBS according to nCounter XT Assay procedures (NanoString, Seattle, WA).

For mRNA analysis, 80,000 live cells were utilized. Gene expression was directly measured via counts of corresponding mRNA CodeSets in each sample using nCounter PanCancer Immune Profiling Panel (NanoString) kit, which is a 770-plex gene and 30-plex protein expression panel measuring human immune response in solid and cancer types [44]. Analysis of raw mRNA and protein counts was performed using nSolver v3.1. For proteins analysis, 160,000 live cells were utilized. Protein expression was directly measured via counts of corresponding protein CodeSets. The raw gene and protein expression data were normalized using negative controls. The arithmetic mean plus 2 standard deviations (SDs) of the internal negative controls was subtracted from the gene expression and the protein expression count to minimize nonspecific mRNA detection. Gene expressions were then normalized using the geometric means of two genes, HLA-G and IRAK2. Protein expressions were then normalized using the geometric means of two protein codesets GITR and CD33. These are known to be relatively invariant in expression levels among colon tissue and cell lines [45]. All targets with average count below background (≤ 25.11 count) were excluded. Spearman correlation was used to visually compare overall gene counts between experimental conditions. Volcano plots, visualizing differential mRNA counts between conditions, indicating significance, fold change and false discovery rate (FDR) were generated using grouped data, presented as normalized log_2_ values, as described by nSolver manual. Correction of *p*-values for FDR was done using the Benjamini-Hochberg method [46]. Scatter plots for mRNA and protein counts were generated using grouped data, presented as normalized log_2_ values, as described by nSolver manual.

## 3 Results

### 3.1 Gene expression (transcriptome) profiling of blood from IBS patients versus controls

**Fig. 1:**
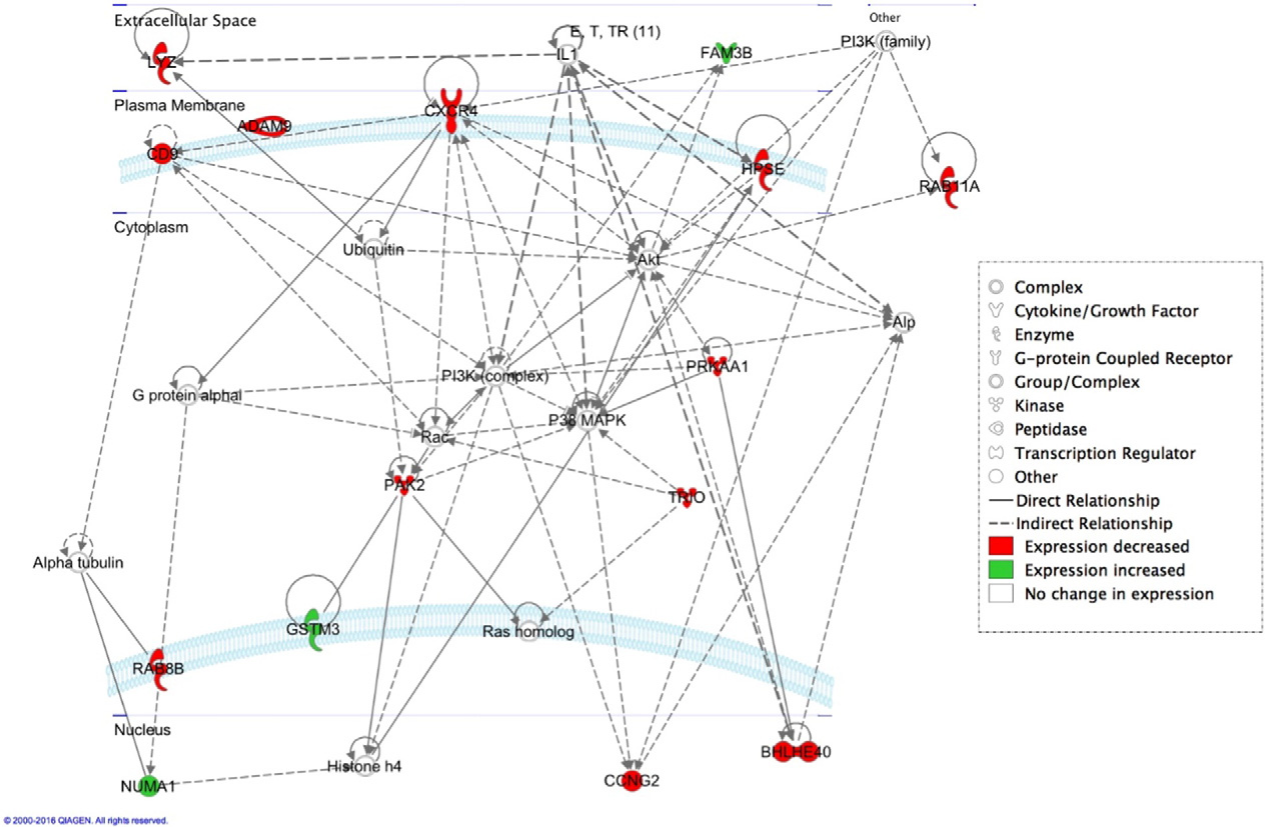
A network generated from gene expression profiling of whole blood from patients with chronic stress-induced GI dysfunction versus controls.

**Table 1:** Demographics and inflammatory marker profiles.

Variable	Overall *n* = 90	Control *n* = 62	IBS *n* = 28	IBS-D *n* = 13	IBS-C *n* = 12	*p*-values*
**Gender n (%)**						
Male	45 (50%)	33 (53.2%)	12 (42.9%)	5 (38.5%)	5 (41.7%)	NS
Female	45 (50%)	29 (46.8%)	16 (57.1%)	8 (61.5%)	7 (58.3%)	NS

**Race n (%)**						
White	45 (50%)	30 (48.4%)	15 (53.6%)	8 (61.5%)	6 (50%)	NS
Black	26 (28.9%)	17 (27.4%)	9 (32.1%)	2 (15.4%)	6 (50%)	NS
Asian	13 (14.4%)	10 (16.1%)	3 (5.2%)	2 (15.4%)	0	NS
Other	6 (6.7%)	5 (8.1%)	1 (3.6%)	1 (7.7%)	0	NS
**Age (yrs)**	27.82 ± 7.85	28.22 ± 8.05	26.93 ± 7.45	26.62 ± 6.99	27.50 ± 8.09	NS
**LBP (**μg**/mL)**	18.69 ± 6.82	18.26 ± 6.10	19.61 ± 8.19	22.78 ± 9.89	16.56 ± 5.33	0.02
**CRP (mg/L)**	2.11 ± 3.12	2.08 ± 3.12	2.18 ± 3.35	2.87 ± 4.47	1.88 ± 2.03	NS
**ESR (mm/h)**	8.71 ± 7.53	8.81 ± 7.86	8.50 ± 6.89	9.15 ± 7.66	9.33 ± 6.39	NS

IBS = Irritable Bowel Syndrome, IBS-D = Irritable Bowel Syndrome- Diarrhea, IBS-C = Irritable Bowel Syndrome- Constipation, CRP = C-reactive protein, ESR = Erythrocyte sedimentation rate, LBP = Lipopolysaccharide Binding Protein. IBS-Mixed (*n* = 3) were excluded from analysis of overall IBS group (*n* = 28). *P-values for LBP are IBS-D compared to control, other comparisons were not significant (NS).

**Table 2:** Signaling network and biological pathways (IPA; DAVID) generated by blood transcriptome of patients versus controls.

Top canonical pathways (IPA)		*p*-Value
Pyridoxal 5′-phosphate salvage pathway		0.00187
Salvage pathways of pyrimidine ribonucleotides		0.00407
Atherosclerosis signaling		0.00522

Molecular and cellular functions (IPA)	Genes, n	*p*-Value
Cellular growth and proliferation	13	0.0000471–0.0224
Cellular function and maintenance	12	0.0000150–0.0241
Cellular movement	11	0.0000041–0.0249
		
Physiological system development and function (IPA)	Genes, n	*p*-Value
Hematological system development and function	11	0.0000018–0.0249
Tissue development	11	0.0000649–0.0241
Immune cell trafficking	10	0.0000018–0.0249

Biological processes (DAVID)	Genes, n	*p*-Value
Response to external stimulus	8	0.00456
Intracellular signal transduction	8	0.0121
Regulation of molecular function	8	0.0177
		
Cellular components (DAVID)	Genes, n	*p*-Value
Extracellular region part	12	0.000528
Extracellular region	12	0.00261
Extracellular exosome	11	0.000180
		
Molecular functions (DAVID)	Genes, n	*p*-Value
Enzyme binding	6	0.0299
Carbohydrate derivative binding	6	0.0704
Protein serine/threonine kinase activity	3	0.0790

### 3.2 Characterization of plasma EVs from IBS patients and controls

**Fig. 2:**
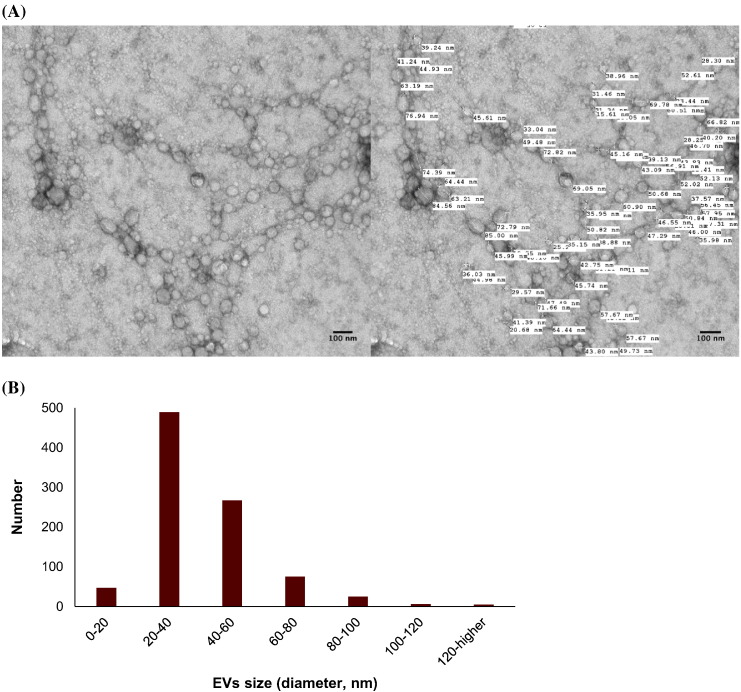

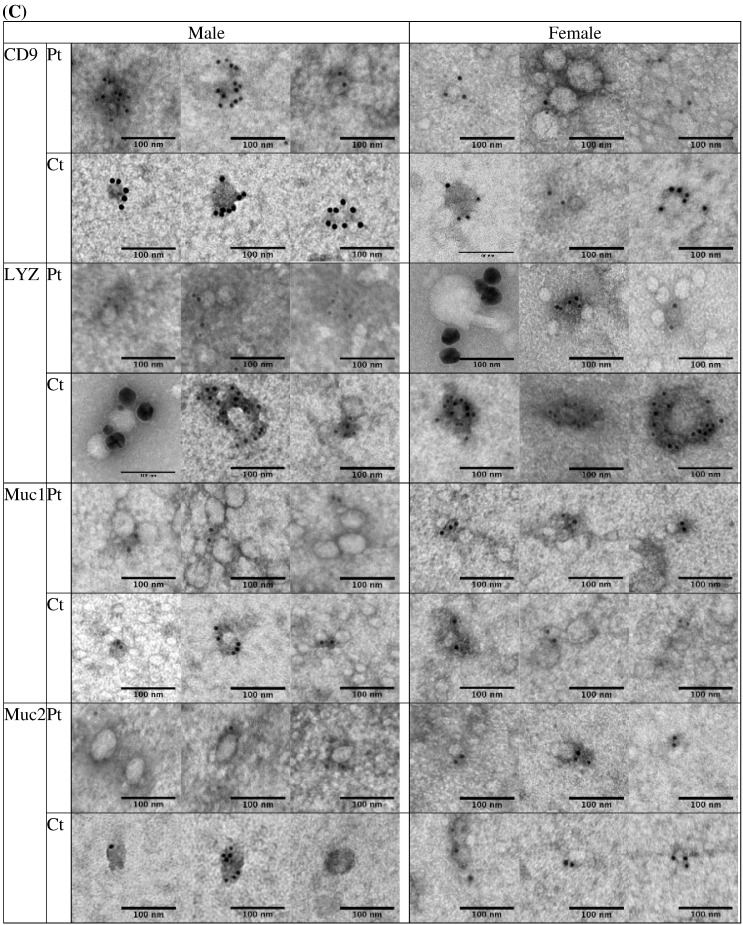
(A) EVs isolated using size exclusion chromatography and detection by uranyl acetate negative staining followed by transmission electron microscopy visualization. Example of EV diameter size quantification by transmission electron microscopy. (B) A histogram of EV size distribution from a representative pool of 914 EVs from patients and controls. (C) Plasma EVs from patients (Pt) and controls (Ct) showed immunogold-labelling with CD9, lysozyme (LYZ), mucin 1 (MUC1), and mucin 2 (MUC2) by transmission electron microscopy. All scale bars are 100 nm.

### 3.3 Lysozyme induced cell migration and accelerated scratch wound repair

**Fig. 3:**
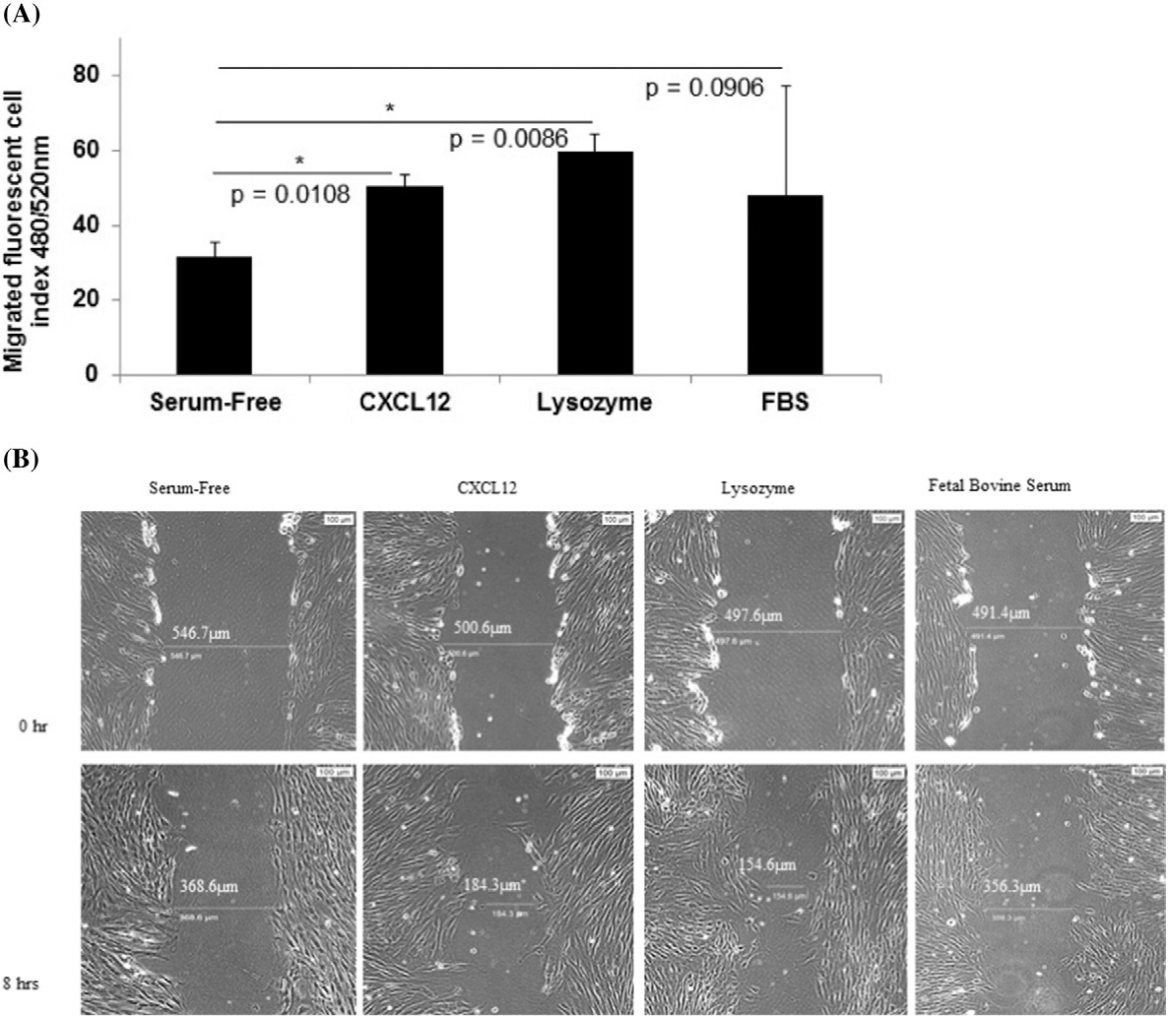
(A) Human fetal colon epithelial cells CRL-1790 migrated towards lysozyme-containing media. Negative control was serum-free media (“Serum-Free”). Positive control was CXCL12-containing media. Fluorescent index values are means ± standard deviations from three biological replicates. (B) Scratch wound assay: Following an overnight incubation in serum-free media (serum starvation), CRL-1790 cells were scratch-wounded at time 0 h followed by incubation in serum-free media containing lysozyme, CXCL12, or 10% fetal bovine serum. Negative control was serum-free media. Images were taken and wound gaps were measured at 0 h and 8 h post-wounding.

### 3.4 Altered immune-linked mRNA and cellular protein levels induced by lysozyme in response to wound and serum starvation

**Fig. 4:**
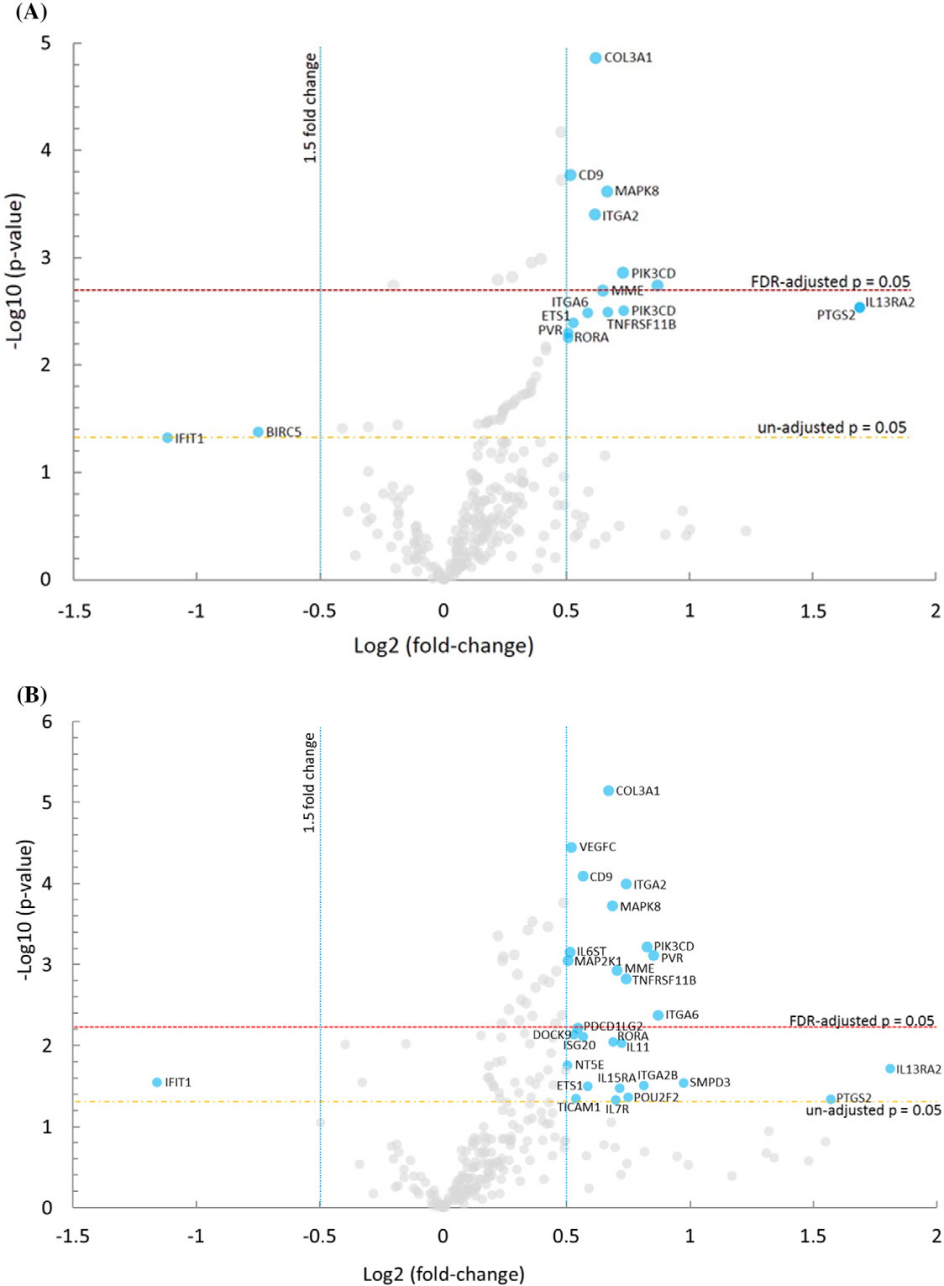

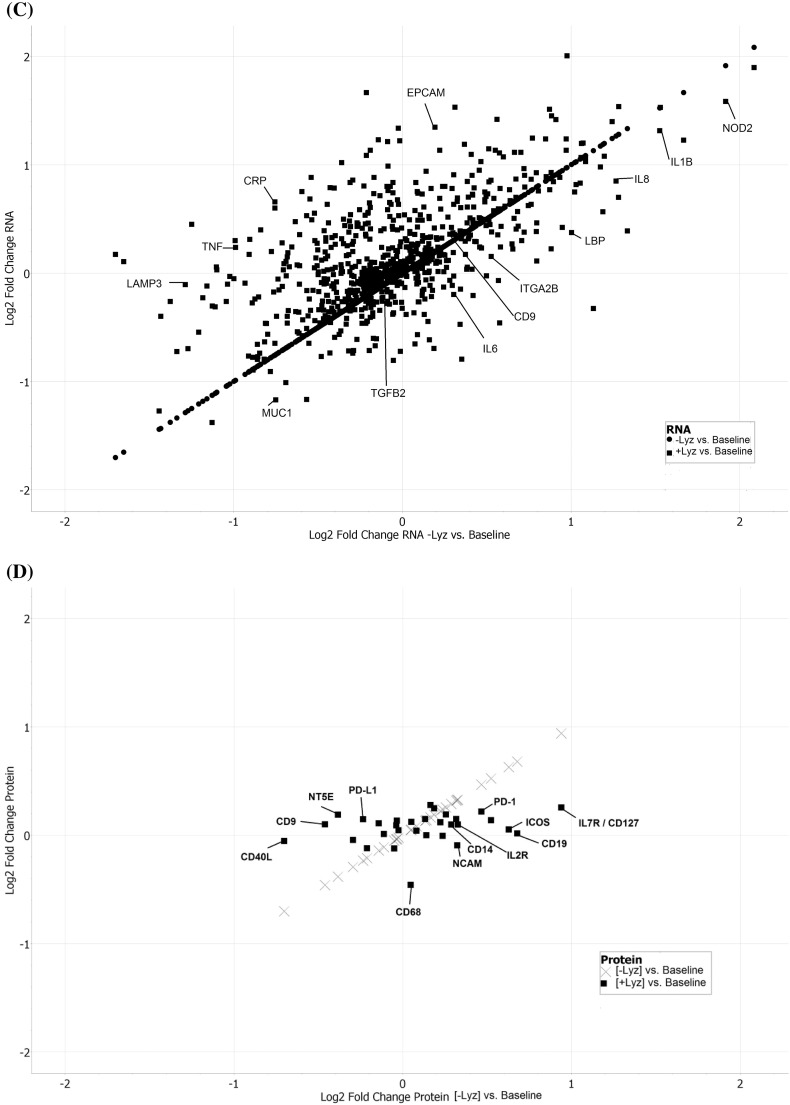

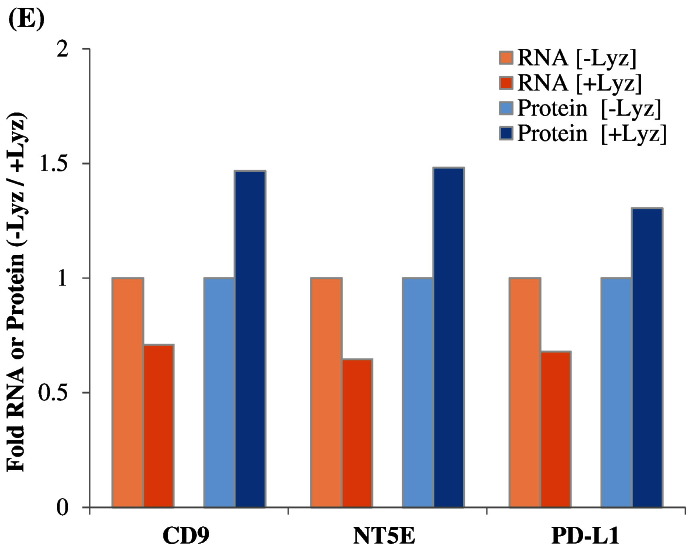
Results of the nCounter RNA:Protein Immune Panel analyses of cellular alterations in response to serum starvation and scratch wound, in the presence or absence of lysozyme, are plotted. In (A), a volcano plot shows differential mRNA levels in CRL-1790 cells surviving at 8 h post-wounding in the presence of lysozyme (+ Lyz) versus in cells harvested at 0 h post-wounding (Baseline). In (B), a volcano plot shows differential mRNA levels in cells surviving at 8 h post-wounding in the absence of lysozyme (− Lyz) versus baseline. In (C), all genes whose mRNA levels were altered in the presence of lysozyme (+ Lyz) versus baseline were plotted against all genes whose mRNA levels were altered in the absence of lysozyme (− Lyz) versus baseline were plotted, regardless of their significance ranking (*p*-values). (D) Immune-related proteins whose levels were altered 8 h post-wounding in the presence (+ Lyz) or absence (− Lyz) of lysozyme, both compared to Baseline, are shown. (E) Lysozyme-dependent alterations of three highest expressed immune-linked proteins and their corresponding mRNAs levels in CRL-1790 cells which survived at 8 h post-wounding are shown.

**Table 3:** Top 20 genes ranked by highest significance (*p*-values) for dysregulation in lysozyme-treated, surviving cells at 8 h post-wounding (+ Lyz), compared to baseline at 0 h.*

	Log2 fold change	Lower confidence limit	Upper confidence limit	*P*-value	FDR
COL3A1	0.619	0.476	0.761	1.37E-05	0.0219
VEGFC	0.479	0.344	0.615	6.70E-05	0.0532
CD9	0.515	0.351	0.679	0.000169	0.0745
PDGFC	0.482	0.326	0.638	0.000188	0.0745
MAPK8	0.664	0.442	0.886	0.000243	0.0773
ITGA2	0.616	0.395	0.837	0.000394	0.104
*ITGB3	0.397	0.234	0.561	0.00102	0.218
TICAM2	0.359	0.21	0.508	0.0011	0.218
PIK3CD	0.731	0.416	1.05	0.00138	0.233
IRAK1	0.28	0.158	0.402	0.0015	0.233
MFGE8	0.221	0.123	0.318	0.00161	0.233
*PSMB8	-0.201	-0.291	-0.111	0.00181	0.24
MME	0.647	0.351	0.943	0.00203	0.248
*CSF2RB	0.734	0.379	1.09	0.00287	0.288
*TNFRSF11B	0.667	0.344	0.991	0.0029	0.288
APP	0.231	0.118	0.344	0.0031	0.288
*ILF3	0.331	0.168	0.494	0.00322	0.288
*IKBKG	-0.411	-0.614	-0.208	0.00326	0.288
CD63	0.252	0.123	0.38	0.00405	0.338
*CD276	0.287	0.135	0.439	0.00497	0.395

⁎ Marks genes whose mRNA expressions were uniquely altered in the presence of lysozyme.

**Table 4:** Top 20 genes ranked by highest significance (*p*-values) for dysregulation in non-lysozyme-treated, surviving cells at 8 h post-wounding (− Lyz), compared to baseline at 0 h.

	Log2 fold change	Lower confidence limit	Upper confidence limit	*P*-value	FDR
COL3A1	0.669	0.526	0.812	7.26E-06	0.0115
VEGFC	0.52	0.384	0.655	3.58E-05	0.0285
CD9	0.567	0.403	0.731	8.16E-05	0.0407
ITGA2	0.74	0.519	0.961	0.000102	0.0407
PDGFC	0.488	0.332	0.644	0.000172	0.0502
MAPK8	0.687	0.465	0.909	0.00019	0.0502
NFATC2	0.36	0.237	0.484	0.000294	0.0658
TICAM2	0.425	0.276	0.574	0.000341	0.0658
IRAK1	0.343	0.221	0.465	0.000373	0.0658
HMGB1	0.221	0.14	0.301	0.000442	0.0702
PIK3CD	0.827	0.512	1.14	0.000605	0.0842
IL6ST	0.513	0.313	0.713	0.000703	0.0842
APP	0.287	0.174	0.4	0.000754	0.0842
PVR	0.851	0.515	1.19	0.000772	0.0842
CD164	0.243	0.146	0.339	0.000805	0.0842
MAP2K1	0.506	0.302	0.711	0.000901	0.0842
MFGE8	0.241	0.144	0.338	0.000901	0.0842
MME	0.704	0.408	1	0.00118	0.101
CD97	0.458	0.265	0.651	0.00121	0.101
CD63	0.301	0.172	0.43	0.00133	0.105

The nCounter RNA:Protein Immune Panel allows comparison of the expression levels of 30 proteins with their corresponding mRNA levels. Analysis of proteins versus mRNAs with post-normalized counts > 15 found three proteins whose expressions were altered in the opposite direction (upregulated versus downregulated) compared to their corresponding mRNAs, in cells incubated with or without lysozyme post-wounding (Fig. 4E). These three proteins and corresponding mRNAs were CD9, NT5E, and PD-L1 (Fig. 4E).

## 4 Discussion

Here we describe first-time evidence of the presence of lysozyme on the surface of human plasma EVs. We further demonstrate the importance of lysozyme for epithelial cell migration and find that lysozyme treatment alters the expression of a number of proteins in cells relevant to inflammatory and immune signaling. Blood gene expression (transcriptome) profiling of IBS patients compared to healthy controls reveals a signaling network of the pyridoxal 5′ phosphate salvage pathway, salvage pathway of pyrimidine ribonucleotides, and atherosclerosis pathway. These pathways suggest the involvement of inflammation in stress pathophysiology. In addition, molecular pathways of cell-to-cell signaling and interaction, cellular movement, and cellular assembly and organization are predicted. Network illustration (IPA) suggests several upstream effectors, including CD9 and lysozyme. DAVID analysis additionally reveals pathways related to extracellular exosomes. Because of increasing reports of EVs acting as carriers for critical effectors of cellular signaling pathways including those involved in GI diseases [11], [12], plasma EVs from representative patients and controls were investigated. Intact plasma EVs from patients and controls exhibited labelling with CD9, mucin 1, mucin 2, and lysozyme proteins. Although lysozyme was recently shown to be associated with human urine EVs [20], the present study shows the characterization and association of lysozyme proteins with plasma EVs. To investigate the potential role of lysozyme as an effector of signaling, we performed *in vitro* functional studies testing the effects of lysozyme on cell migration, which was identified among the top pathways by transcriptome profiling. In human primary colorectal cell line, CRL-1790, we found that cell migration in response to wound, introduced after an overnight serum starvation, was observed when lysozyme was present compared to when lysozyme was absent in cell media. This provides functional evidence for role of lysozyme in cellular response to wound or injury. Further, cells that survived wounding were subjected to a global RNA and protein profiling to examine lysozyme-specific endogenous alterations in immune signaling. Lysozyme treatment induces altered expression of mRNAs and proteins in surviving cells, including CD9, NT5E, PD-L1, IL6, IL8, and IL1B. These proteins have been shown by others to be involved during early and late phase wound repair, cell migration, inflammation, and regeneration [1], [2], [6]. Our data suggest that lysozyme may be part of the underlying pathophysiology for the chronic stress-exacerbated GI symptoms observed in IBS patients.

Lysozyme's co-localization in mouse intestine [27] with LBP, an inflammatory marker found in obesity and other metabolic conditions [55], supports the potential role of lysozyme in inflammation. Our nCounter analyses illustrate the downstream effects of lysozyme inimmune-linked signaling in human colon epithelial cells which survive following scratch wounding. The mRNAs and proteins which are altered by the addition of lysozyme to the cells include inflammatory related molecules (eg., IL1B, CD9, see Data in Brief). Some of these, such as CD9, has been shown by others to be altered during stress [2]. Other mRNAs correspond to the Unfolded Protein Response/ Endoplasmic Reticulum (ER) stress response, such as the lysosomal-associated membrane protein or LAMP [56], [57], to be differentially expressed in the presence verses absence of lysozyme. Future studies shall elucidate pathways in which these genes may act synergistically to elicit cellular responses in the context of stress and inflammation.

The specific involvement of lysozyme in cellular signaling needs to be considered in the context of IBS. It is possible that reduced levels of circulating lysozyme RNA translate to an inability to respond to injury in IBS patients, as levels of circulating RNAs have been shown to be related to post-injury state in animal models [14]. Other explanations may include that lower lysozyme levels are due to lack of baseline innate immune response or over-utilization due to microbial translocation as a result of microbial dysbiosis. Gut bacteria populations in IBS patients may potentially contribute to the observed differential appearance of lysozyme in the circulation. To this end, we recently reported alterations in microbial composition and abundance in IBS patients versus controls [38]. We showed that IBS-overweight patients had decreased richness in the phylum Bacteroidetes (*p* = 0.007) and the genus *Bacillus* (*p* = 0.008) compared to healthy individuals. Further, when patients drank a four-sugar probe intestinal permeability test solution, which induced abdominal pain, post-ingestion abdominal pain was most severe in IBS patients, particularly those who were overweight. Thus, the microbiome characteristics may influence lysozyme's association with plasma EVs in IBS patients and healthy controls, especially given that lysozyme exerts immune function by binding to bacteria cell walls [18].

While the nCounter RNA:Protein Immune Panel has the advantages of multiplexing hundreds of gene targets in a single reaction, limitations include the non-spatial resolution of data, with counts representing average from the entire cell populations [44]. Thus, the signals reported herein were average signals from wounded and non-wounded cells as well as migrating and non-migrating cells. Also, because the probe-sets were pre-designed, the data generated reflected targeted discovery instead of pure discovery [44]. Nonetheless, the ability to simultaneously probe for proteins and their corresponding mRNAs provides a powerful tool to screen for post-transcriptional and/or post-translational regulation for a particular gene. In our system, CD9, NT5E, and PD-L1 protein levels were upregulated, while mRNA levels were downregulated, in the presence or absence of lysozyme at 8 h post-wounding (Fig. 4E). The upregulated protein expression could be due to either increased protein stability (e.g., reduced protein degradation by ubiquitin) or increased *de novo* translation, whereas downregulated mRNA expression could be due to either increased mRNA degradation (e.g., by miRNA) or decreased *de novo* transcription (e.g., for CD9, reduced CD9 promoter activation by TCF/LEF transcription factor) in the presence of lysozyme. To explore these mechanisms, one potential test is kinetic experiments of the involved regulatory components using similar multiplex panels focusing on miRNAs.

In conclusion, we demonstrate the potential role of lysozyme in colon epithelial cell migration, cellular response to wound, and show its association with chronic stress related symptoms in IBS patients. Our study identifies lysozyme to be associated with intact plasma EVs, suggesting their involvement in cell-cell communication. Cell-cell communication signals described herein may be exploited for studies of the pathophysiology of stress-induced intestinal damage [58], with the ultimate goal of future utilization in repairing GI barrier damage.

## Conflict of interest and funding

We acknowledge the funding from the US Department of Health and Human Services, National Institutes of Health, National Institute of Nursing Research, Division of Intramural Research (1ZIANR000018; PI, WAH; Intramural Training Awards to PVJ, LBS, NHF, PAS, KMB, KRW) and the National Cancer Institute, under Contract No. HHSN261200800001E. No other funding or benefits from industry or elsewhere were received to conduct this study.

## Ethical principles

The study was approved by the NIH Institutional Review Board and complied with the Declaration of Helsinki with voluntary written informed consent from all participants included in the study (clinicaltrials.gov #NCT00824941). The opinions expressed herein and the interpretation and reporting of these data are the responsibility of the author(s) and should not be seen as an official recommendation, interpretation, or policy of the National Institutes of Health or the United States Government.

## Author contributions

SKA and WAH initiated the hypothesis of the study and designed the protocol. YY and SAK performed the initial vesicle experiments and PVJ, CMB, NDK and KRW assisted with technical and biological replicated of the experiments. SKA, NHF, GEG completed the NanoString experiments and analysis. SKA, PVJ, LBS, WAH, PAS, and KRW completed the clinical data and analysis. CKEB and ESS assisted with electron microscopy methods and analysis. All of the authors contributed to the generation and editing of the final manuscript.

## Transparency Document

Transparency document Image 1

## Appendix A Supplementary data

Supplementary material Image 2
