# Supplementary material for: Lysozyme association with circulating RNA, extracellular vesicles, and chronic stress
Source: BBA Clin. 2016 Dec 20;7:23–35. doi: 10.1016/j.bbacli.2016.12.003 (PMC5200883; doi:10.1016/j.bbacli.2016.12.003)
Supplement: Supplementary file 2 — Supplementary material [file mmc2.docx]

**Appendix A. Supplementary data**

**

**

**
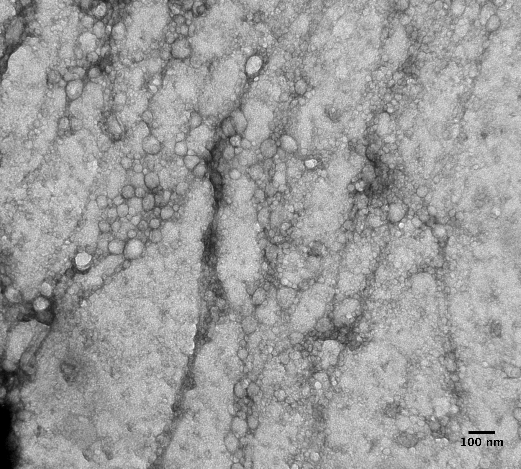
**

**IgG1 IgG2a**

**Fig. S1.** Plasma EVs from patients and controls showed immunogold control labelling with IgG1and IgG2a by transmission electron microscopy are shown. All scale bars are 100 nm.


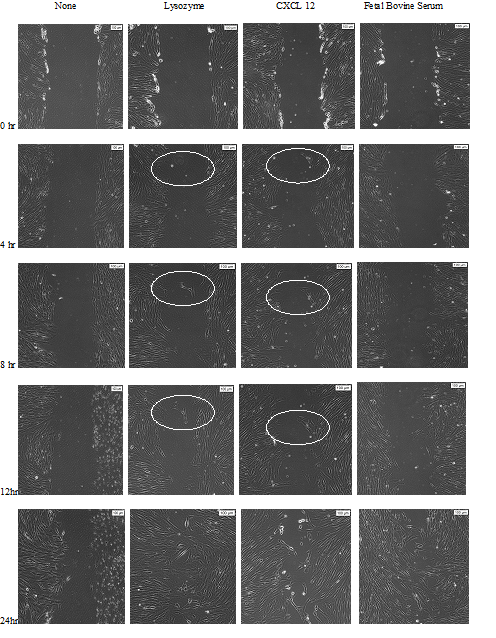


**Fig. S2.** Scratch wound assay: Following an overnight incubation in serum-free media (serum starvation), CRL-1790 cells were scratch-wounded at time 0 hr followed by incubation in serum-free media containing non, lysozyme, CXCL12, or 10% Fetal Bovine Serum. Images were taken over 24 hrs post-wounding.

**Table S1.** Global gene expression profiling (transcriptome) of patients versus controls.

| **Gene Symbol** | **Gene Title** | **p-value** | **Controls (mean)** | **IBS (mean)** | **Fold Change Controls vs. IBS** |
| --- | --- | --- | --- | --- | --- |
| NUMA1 | nuclear mitotic apparatus protein 1 | 0.014442 | 5.96089 | 6.87458 | 1.88385 |
| GSTM3 | glutathione S-transferase mu 3 (brain) | 0.000251 | 7.56729 | 8.34482 | 1.71419 |
| FAM3B | family with sequence similarity 3, member B | 0.045587 | 5.91358 | 6.55486 | 1.55972 |
| ALDH8A1 | aldehyde dehydrogenase 8 family, member A1 | 0.002384 | 5.84843 | 6.43619 | 1.5029 |
| PAK2 | p21 protein (Cdc42/Rac)-activated kinase 2 | 0.010213 | 11.2874 | 10.7012 | -1.50131 |
| PRKAA1 | protein kinase, AMP-activated, alpha 1 catalytic | 0.004346 | 5.40333 | 4.81224 | -1.50638 |
| TRIO | triple functional domain (PTPRF interacting) | 0.008392 | 7.80001 | 7.20758 | -1.50778 |
| CCNG2 | cyclin G2 | 0.018049 | 7.768 | 7.17556 | -1.50779 |
| LOC100506748 | hypothetical LOC100506748 | 0.011536 | 7.56244 | 6.96877 | -1.50908 |
| MTPN | Myotrophin | 0.017795 | 8.98379 | 8.37715 | -1.52271 |
| RAB11A | RAB11A, member RAS oncogene family | 0.016969 | 8.82283 | 8.20562 | -1.53391 |
| P2RY12 | purinergic receptor P2Y, G-protein coupled, 12 | 0.000451 | 6.39976 | 5.77026 | -1.54703 |
| RAB8B | RAB8B, member RAS oncogene family | 0.037204 | 7.70295 | 7.0611 | -1.56033 |
| BOD1L | biorientation of chromosomes in cell division 1-like | 0.004998 | 8.3922 | 7.74379 | -1.56744 |
| CXCR4 | chemokine (C-X-C motif) receptor 4 | 0.047873 | 9.90926 | 9.25646 | -1.57221 |
| HPSE | Heparanase | 0.000132 | 9.28654 | 8.63375 | -1.57221 |
| CD9 | CD9 molecule | 0.018062 | 8.79324 | 8.11997 | -1.59469 |
| ADAM9 | ADAM metallopeptidase domain 9 | 0.001717 | 7.71891 | 7.03697 | -1.6043 |
| LYZ | Lysozyme | 0.011793 | 11.2726 | 10.5673 | -1.63052 |
| BHLHE40 | basic helix-loop-helix family, member e40 | 0.00166 | 8.91057 | 8.17266 | -1.66775 |
